# Propagule Pressure, Habitat Conditions and Clonal Integration Influence the Establishment and Growth of an Invasive Clonal Plant, *Alternanthera philoxeroides*

**DOI:** 10.3389/fpls.2016.00568

**Published:** 2016-05-03

**Authors:** Wen-Hua You, Cui-Min Han, Long-Xiang Fang, Dao-Lin Du

**Affiliations:** Institute of Environment and Ecology, College of the Environment and Safety Engineering, Jiangsu UniversityZhenjiang, China

**Keywords:** alligator weed, intraspecific interaction, interspecific interaction, plant invasion, propagule supply

## Abstract

Many notorious invasive plants are clonal, spreading mainly by vegetative propagules. Propagule pressure (the number of propagules) may affect the establishment, growth, and thus invasion success of these clonal plants, and such effects may also depend on habitat conditions. To understand how propagule pressure, habitat conditions and clonal integration affect the establishment and growth of the invasive clonal plants, an 8-week greenhouse with an invasive clonal plant, *Alternanthera philoxeroides* was conducted. High (five fragments) or low (one fragment) propagule pressure was established either in bare soil (open habitat) or dense native vegetation of *Jussiaea repens* (vegetative habitat), with the stolon connections either severed from or connected to the relatively older ramets. High propagule pressure greatly increased the establishment and growth of *A. philoxeroides*, especially when it grew in vegetative habitats. Surprisingly, high propagule pressure significantly reduced the growth of individual plants of *A. philoxeroides* in open habitats, whereas it did not affect the individual growth in vegetative habitats. A shift in the intraspecific interaction on *A. philoxeroides* from competition in open habitats to facilitation in vegetative habitats may be the main reason. Moreover, clonal integration significantly improved the growth of *A. philoxeroides* only in open habitats, especially with low propagule pressure, whereas it had no effects on the growth and competitive ability of *A. philoxeroides* in vegetative habitats, suggesting that clonal integration may be of most important for *A. philoxeroides* to explore new open space and spread. These findings suggest that propagule pressure may be crucial for the invasion success of *A. philoxeroides*, and such an effect also depends on habitat conditions.

## Introduction

Plant invasion has posed a great threat to biodiversity, environment, and economy both globally and locally (Mack et al., 2000; Vila et al., 2011). Previous studies have demonstrated that plant invasion is the outcome of complicated interactions that involve many biotic and abiotic factors (Davis et al., 2000; Lockwood et al., 2005; Melbourne et al., 2007; Chun et al., 2010), which can be divided into three broad categories: propagule pressure (the number of propagules entering the new habitat), invasibility of the environment (habitat conditions) and the characteristics of the plant species (such as clonal traits; Davis et al., 2000; Simberloff, 2009; Ehrenfeld, 2010; Xie et al., 2013).

Propagule pressure (propagule supply) has been considered one of the most important factors for explaining the invasion success of plants (Levine and D’Antonio, 1999; Britton-Simmons and Abbott, 2008; Simberloff, 2009; Duncan, 2011). It has been proposed that the greater number of propagules arriving in a new environment gives a plant a higher chance to establish itself, persist, naturalize, spread, and invade (Rouget and Richardson, 2003; Lockwood et al., 2009; Simberloff, 2009). Indeed, many previous studies have showed a positive relationship between propagule pressure and invasion success of introduced plants (Rouget and Richardson, 2003; Colautti et al., 2006; Lockwood et al., 2009; Liu et al., 2014). Despite its acknowledged importance, propagule pressure has rarely been experimentally studied (but see Roiloa and Retuerto, 2005), and the interaction of propagule pressure with other factors (such as disturbance and habitat conditions) that influence invasion success is still not well understood (Lockwood et al., 2005; Britton-Simmons and Abbott, 2008; Liu et al., 2014).

According to the “ecological resistance hypothesis,” resident native communities may indirectly control invasion success by reducing the input of propagules and resource availability, thereby inhibiting the establishment and spreading process of the introduced species (Levine et al., 2004; Xie et al., 2013). Therefore, the role of propagule pressure in shaping the invasion process of introduced plants may be closely related to habitat conditions (Rouget and Richardson, 2003; Warren et al., 2012; Liu et al., 2014). In habitats where resident native vegetation is scare and much space is available (open habitats), introduced plants may need a few propagules to ensure establishment and invasion success. On the contrary, introduced plants may need a larger number of propagules to overcome high interspecific competition and establish successfully in habitats where resident native vegetation is dense (D’Antonio et al., 2001). However, to our knowledge, relatively few experimental researches have investigated how habitat conditions affect the role of propagule pressure in the invasion process of alien invasive species, although several studies have addressed that the effects of propagule pressure on the invasion success of plants also depended on habitat suitability (or habitat conditions; Holle and Simberloff, 2005; Warren et al., 2012; Liu et al., 2014).

Another important factor for invasion success is the characteristics of the plant species such as clonal traits (Kolar and Lodge, 2001). As we know, many of the most notorious alien invasive plants have the capacity for vigorous clonal propagation (Kolar and Lodge, 2001; Xu et al., 2010; You et al., 2013). For instance, *Eichhornia crassipes* (water hyacinth), *Alternanthera philoxeroides* (alligator weed), and *Myriophyllum aquaticum* (parrotfeather) can grow and spread mainly by vegetative growth and clonal propagation (no seeds or seed sterility) in their introduced regions (Villamagna and Murphy, 2010; Schooler, 2012; Xie et al., 2013). Recently, some studies have pointed out that the invasiveness of alien clonal plants may be closely related to clonal traits such as clonal integration (i.e., the reciprocal translocation of resources between interconnected ramets; Wang et al., 2008; Song et al., 2013; You et al., 2014a). Clonal integration, driven by the source-sink relationship, can improve plants’ exploitation of ubiquitous heterogeneous resources, help plants invade new environments and facilitate plants’ spatial occupation of new habitats for both native and invasive clonal plants (Klimeš et al., 1997; Yu et al., 2009). For example, clonal integration may increase competitive ability of invasive plants when grown with resident native vegetation, thereby influence species co-existence, community structure, and ecosystem functioning (Yu et al., 2009; You et al., 2014a). Although several studies have addressed that clonal integration had positive effects on establishment and growth of the invasive clonal plants (such as *A. philoxeroides* and *Carpobrotus edulis*) in different habitat conditions (bare soil or vegetation; Wang et al., 2008; Yu et al., 2009; Roiloa et al., 2010; You et al., 2014a), unfortunately, all these studies ignored the role of propagule pressure in shaping this process with regard to vegetative propagules.

*Alternanthera philoxeroides*, originating from the Parana River region of South America, is a clonal weed that causes serious economic and environmental problems worldwide (Julien et al., 1995; Schooler, 2012). It is stoloniferous and amphibious, growing in both riparian and terrestrial habitats (Schooler, 2012). This species is one of the world’s worst invasive weeds and is listed as one of the 16 worst alien invasive weeds in China (Julien et al., 1995; Ma and Wang, 2005). *A. philoxeroides* often suffers natural disturbances, such as herbivory, mowing and trampling, which may fragment its clones into pieces (Schooler et al., 2007; Dong et al., 2010, 2012; You et al., 2014b). In China, *A. philoxeroides* has extremely low genetic diversity (Xu et al., 2003; Wang et al., 2005), and clonal integration plays an important role in determining its growth and spread (Wang et al., 2008, 2009; Xu et al., 2010; You et al., 2014b). *Jussiaea repens* is a rooted emergent stoloniferous clonal plants and a fast-proliferating species in wetlands, naturally distributed in central and south China. In natural environments, these two species often co-exist in aquatic habitats or aquatic-terrestrial ecotones in south China (You et al., 2014a).

To investigate the effects of propagule pressure, habitat conditions and clonal integration on the establishment, growth and thus invasion success of introduced invasive clonal plants, we selected these two co-occurring stoloniferous clonal plants, *A. philoxeroides* (invasive) and *J. repens* (native). In an 8-week greenhouse experiment, we grew one fragment (low propagule pressure) or five fragments (high propagule pressure) of *A. philoxeroides* either in bare soil (open habitat) or dense native vegetation of *J. repens* (vegetative habitat), with the stolon connections either severed from or connected to the older ramets to test the effect of clonal integration. Specifically, we test the following hypotheses. (1) Increase in propagule supply will increase the establishment and growth of *A. philoxeroides*, especially when it grew in vegetative habitats. (2) Clonal integration will promote the growth and competitive ability of *A. philoxeroides* under high propagule pressure. (3) High propagule pressure of *A. philoxeroides* with clonal integration will reduce the growth of *J. repens*.

## Materials and Methods

### Plant Material

Given that genetic diversity of wetland clonal plants is relatively low (Sosnová et al., 2011), especially for *A. philoxeroides* in China (Xu et al., 2003; Wang et al., 2005), source material of *A. philoxeroides* and *J. repens* were collected in middle June 2014, from at least five locations at least 20 m apart in each of two wetlands in Gonghu Bay of the Taihu Lake in the Jiangsu province of China (N 31°25′–31°28′, E 120°15′–120°21′). Then plants from different locations were mixed and propagated in the greenhouse. After 2 weeks of adaptive culture, about 200 tip cuttings of *A. philoxeroides* and about 1000 tip cuttings of *J. repens* were selected and planted vertically into 20 plots (30 cm diameter × 15 cm height) with lake soil (Total nitrogen concentration 3.05 mg g^−1^, total phosphorus concentration 0.16 mg g^−1^) for continued culture.

### Experimental Design

**FIGURE 1:**
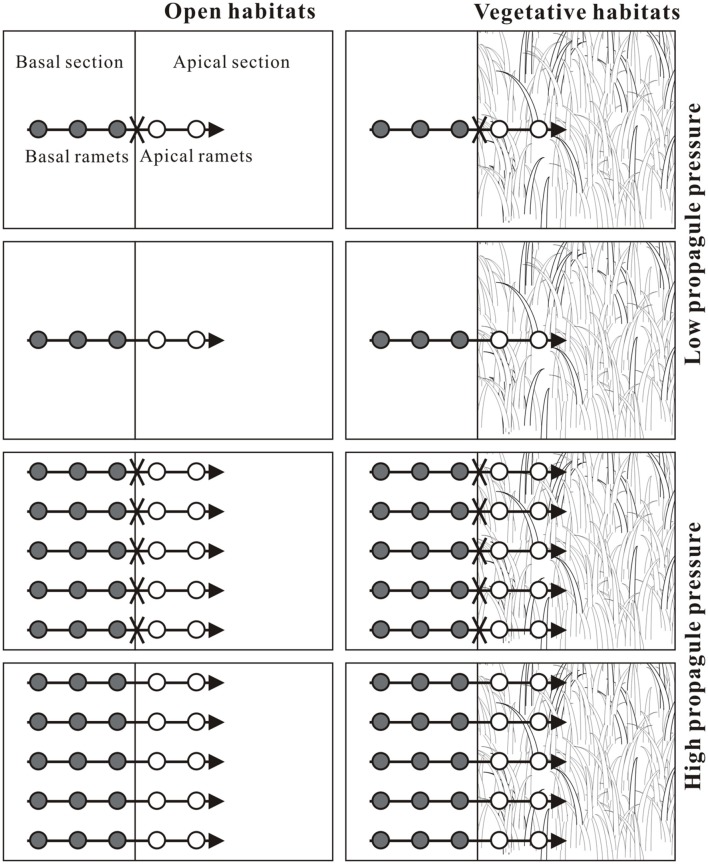
**Schematic representation of the experimental design.** One clonal fragment (low propagule pressure) or five clonal fragments (high propagule pressure) of the invasive plant *Alternanthera philoxeroides*, each consisting of three basal ramets (dark gray circles) and two apical ramets (white circles) with a stolon apex (horizontal arrow), were grown either in open habitats or in vegetative habitats (*Jussiaea repens*), and with stolon connections between basal and apical ramets were either intact or severed (fork).

The growth experiment was conducted in a greenhouse under natural sunlight (about 14/10 day/night cycle) and ambient temperature at the Field Station of Jiangsu University. The experiment was conducted with a factorial design involving propagule pressure (low or high; i.e., one fragment or five fragments), habitat conditions (open or vegetative) and clonal integration (stolon connections were severed or intact; **Figure 1**). The tested plants used in this experiment were 120 similar-sized clonal fragments of *A. philoxeroides* (tip cuttings, 15.27 ± 0.20 cm in length, 0.51 ± 0.07 g in dry mass; means ± SE, measured by another 20 clonal fragments), each consisting of a stolon with five ramets. No differences between treatments were detected in initial size of this plants (*P* > 0.05, One-way ANOVA). Each clonal fragment was divided into two parts, one termed as ‘basal part’ consisting of three relatively old ramets (close to the mother ramets) and the other as ‘apical part’ consisting two relatively young ramets (distal to the mother ramets) and a stolon apex (You et al., 2014a).

There were 45 plastic containers (50 cm × 50 cm × 25 cm; length × width × height), each having two separated sections in this experiment (see **Figure 1**). The basal section was 20 cm long and the apical section was 30 cm long. Resources (nutrients and water) and roots in the two sections did not interfere with each other. All the containers in both sections were filled with a mixture of sand and lake mud at a volume ratio of 3:1 and with 2.0 g slow-release fertilizer (Osmocote, N–P–K: 16–8–12, 6 months). On July 5th 2014, the apical sections of 25 containers were planted vertically with cultured plant fragments (tip cuttings, 15.05 ± 0.22 cm in length, 0.55 ± 0.05 g in dry mass; means ± SE, measured by another 20 clonal fragments) of *J. repens* (monoculture) in the greenhouse to mimic natural plant populations (vegetative habitats), with a density of 200 plants m^−2^ (30 plants in each apical section; You et al., 2014a). The remaining 20 containers were kept with apical sections bare (open habitats).

After 4 weeks adaptive growth of *J. repens* populations in vegetative habitats, on August 3rd 2014, one (low propagule pressure) or five (high propagule pressure) fragments of *A. philoxeroides* were horizontally positioned in both habitat conditions of 40 containers (20 open habitats and 20 vegetative habitats in apical sections), the remaining five containers with vegetative habitats were used as a control for plant population growth of *J. repens* without competition. For each clonal fragment, three ramets of basal part were placed within basal section of a container and the other two ramets and apex of the apical part were within the apical section of the same container (**Figure 1**). The stolons of basal and apical ramets were both anchored to the soil surface to facilitating rooting. Five days later, when the clonal fragments were successfully rooted, the stolon connections between the apical and basal parts were severed in 20 containers, while the other 20 ones were kept intact. Therefore, each treatment was replicated five times (see **Figure 1**). The experiment was conducted for 8 weeks and ended on October 6th 2014. The experimental containers were randomly repositioned every 2 weeks to avoid the effects of possible environmental heterogeneity (such as light), and watered every other day to keep the soil in the containers wet. The mean light intensity at the top of the plant canopy was 1200–1400 μmol m^−2^ s^−1^ on the cloudless days, and the mean air temperature was 25–30°C during the experimental period.

### Measurements

At the final harvest, the number of ramets and leaves were recorded, and total stolon length of *A. philoxeroides* was measured for the apical sections of all treatments. Then the plants of *A. philoxeroides* in the apical part of the container were harvested and separated into leaves, stolons and roots, and their biomass was determined after drying at 70°C for 72 h. Neighboring vegetation of *J. repens* (entire plants including roots) in the apical sections of each container were also harvested and their dry mass was also determined in the same way.

### Data Analysis

Data were expressed as means ± SE. Growth measures (total biomass, ramet number, leaf number, and stolon length) of *A. philoxeroides* in the apical part were calculated at both individual level and population (container) level. Prior to analysis, data were log-transformed if necessary to meet the assumptions of normality and homoscedasticity.

The intraspecific relative competition intensity (RCI) of *A. philoxeroides* was calculated as intraspecific RCI = (G_low_ –G_high_)/G_low_, where G_low_ is the mean growth measure of *A. philoxeroides* in low propagule pressure and G_high_ is that measure in high propagule pressure (Weigelt and Jolliffe, 2003). The index was calculated for each container and averaged for comparison between the two habitat conditions (open and vegetative) with the stolon connections either severed or intact. The interspecific RCI was calculated as interspecific RCI = (G_open_ –G_vegetative_)/G_open_, where G_open_ is the mean growth measure of *A. philoxeroides* in open habitats and G_vegetative_ is that measure in vegetative habitats (Weigelt and Jolliffe, 2003). The index was also calculated for each container and averaged for comparison between the low and high propagule pressure with the stolon connections either severed or intact. A positive value of RCI suggests competition and a negative one indicates facilitation (Armas et al., 2004; Liu et al., 2014). The RCI of *J. repens* was not considered because a significant competition effect was not observed (see Results).

Three-way ANOVA was used to assess the effects of propagule pressure, habitat conditions and clonal integration on the growth measures of *A. philoxeroides* in the apical section at individual level and container level. Two-way ANOVA was employed to investigate the effects of habitat conditions and clonal integration on intraspecific RCI of *A. philoxeroides*, and the effects of propagule pressure and clonal integration on interspecific RCI of *A. philoxeroides*. One-way ANOVA was used to test whether total biomass of vegetation (*J. repens*) in the apical section differed between the four competition treatments and the control. *Post hoc* pair-wise comparisons of the means were performed to examine differences between the treatments using Studentized Tukey’s HSD for multiple comparisons. Statistical significance was assigned at *P* < 0.05. All data analyses were performed using SPSS 17.0 (SPSS, Chicago, IL, USA).

## Results

### Growth of *A. philoxeroides*

**FIGURE 2:**
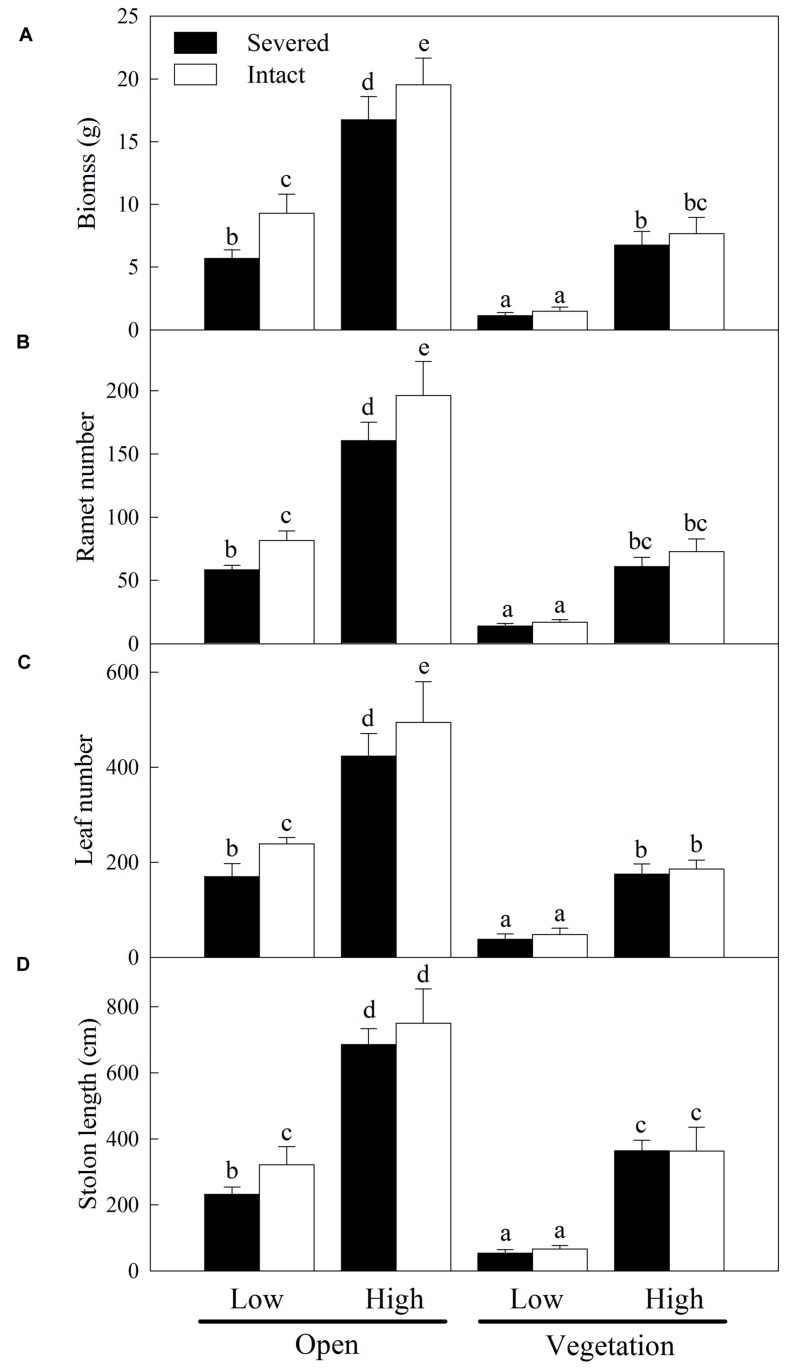
**Effects of experimental treatments on the growth measures of *A. philoxeroides* at container level.** Total biomass **(A)**, ramet number **(B)**, leaf number **(C)**, and stolon length **(D)** of the invasive plant *A. philoxeroides* (low or high propagule pressure) in the apical sections, grown either in open habitats or in vegetative habitats (*J. repens*), and with stolon connections between basal and apical ramets were either intact or severed. Data indicate the means ± SE. Bars sharing the same letter are not significantly different at *P* = 0.05.

At the population (container) level, propagule pressure, habitat condition and clonal integration significantly affected all the growth measures (total biomass, ramet number, leaf number, and stolon length), and propagule pressure × habitat conditions, habitat conditions × clonal integration had also significant effects on growth of *A. philoxeroides* in the apical sections (**Table 1**). High propagule pressure greatly increased the growth of *A. philoxeroides* in both open and vegetative habitats (**Figure 2**), and the effect of propagule pressure on the growth of *A. philoxeroides* was more significant in vegetative habitats than in open habitats (**Figure 2**, 92.7–205.2% of growth increase in open habitats vs. 442–593% of growth increase in vegetative habitats). Moreover, vegetation of *J. repens* significantly suppressed the growth of *A. philoxeroides* in both low and high propagule supply (**Table 1**; **Figure 2**). In open habitats, clonal integration greatly promoted the growth of *A. philoxeroides* either in low propagule supply or in high propagule supply, however, such effect of clonal integration on plant growth disappeared when grown with *J. repens* (**Figure 2**).

**Table 1:** Three-way ANOVA analyses for the effects of propagule pressure, habitat conditions (vegetation) and clonal integration (connection) on the growth measures of the invasive plant *Alternanthera philoxeroides* in the apical sections at container level and individual level.

Dependent variable	Propagule pressure (P)	Vegetation (V)	Connection (C)	P × V	P × C	V × C	P × V × C
**Container level**							
Biomass (g)	400.78^∗∗∗^	428.46^∗∗∗^	21.27^∗∗∗^	33.01^∗∗∗^	0.20	9.55^∗∗^	0.68
Ramet number^1^	439.27^∗∗∗^	474.48^∗∗∗^	23.30^∗∗∗^	55.34^∗∗∗^	1.97	8.12^∗∗^	0.07
Leaf number^1^	364.12^∗∗∗^	332.46^∗∗∗^	11.09^∗∗^	23.40^∗∗∗^	0.4	6.06^∗^	0.05
Stolon length (cm)	282.95^∗∗∗^	285.41^∗∗∗^	6.03^∗^	16.62^∗∗∗^	0.32	4.39^∗^	0.23
**Individual level**							
Biomass (g)	81.66^∗∗∗^	411.16^∗∗∗^	32.01^∗∗∗^	93.73^∗∗∗^	14.95^∗∗^	19.15^∗∗∗^	12.04^∗∗^
Ramet number^1^	211.88^∗∗∗^	626.36^∗∗∗^	51.49^∗∗∗^	170.08^∗∗∗^	11.16^∗∗^	24.20^∗∗∗^	9.11^∗∗^
Leaf number^1^	171.05^∗∗∗^	572.86^∗∗∗^	27.54^∗∗∗^	136.28^∗∗∗^	11.82^∗∗^	15.08^∗∗∗^	6.60^∗^
Stolon length (cm)	65.69^∗∗∗^	372.13^∗∗∗^	14.88^∗∗^	95.61^∗∗∗^	9.01^∗∗^	9.01^∗∗^	4.52^∗^
df	1	1	1	1	1	1	1
Error	32	32	32	32	32	32	32

^1^*Lg, transformed. ^∗^P < 0.05, ^∗∗^P < 0.01, ^∗∗∗^P < 0.001.*

**FIGURE 3:**
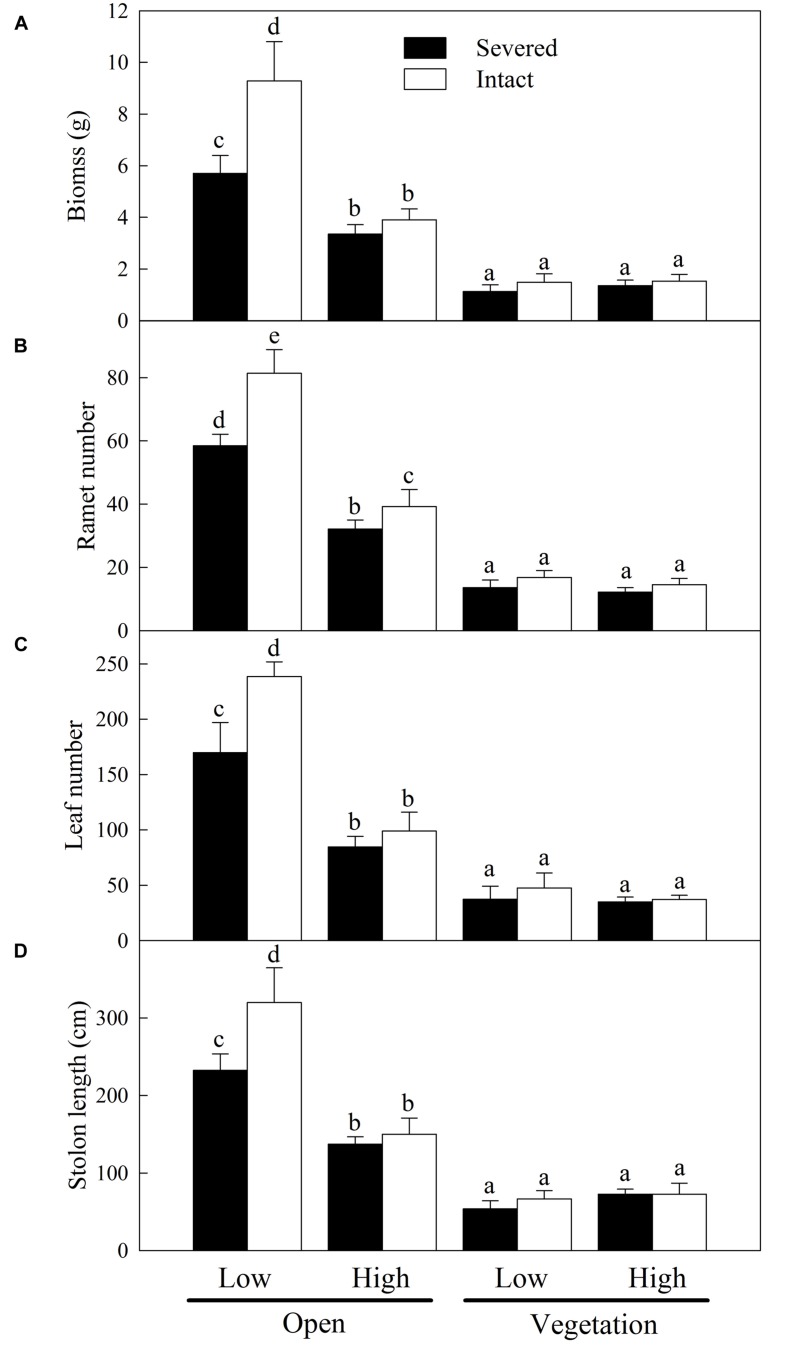
**Effects of experimental treatments on the growth measures of *A. philoxeroides* at initial individual level.** Total biomass **(A)**, ramet number **(B)**, leaf number **(C)**, and stolon length **(D)** of the invasive plant *A. philoxeroides* (low or high propagule pressure) in the apical sections, grown either in open habitats or in vegetative habitats (*J. repens*), and with stolon connections between basal and apical ramets were either intact or severed. Data indicate the means ± SE. Bars sharing the same letter are not significantly different at *P* = 0.05.

At initial individual level, propagule pressure, habitat condition, clonal integration and their interactions had significant effects on the growth of *A. philoxeroides* in the apical sections (**Table 1**). The growth measures of *A. philoxeroides* were greatly higher in open habitats than in vegetative habitats (**Table 1**; **Figure 3**). There were no differences in growth of *A. philoxeroides* among the treatments in vegetative habitats (**Figure 3**). However, in open habitats, the growth measures were significantly lower in high propagule pressure than in low propagule pressure, and clonal integration significantly increased the growth of *A. philoxeroides* only in low propagule pressure (**Figure 3**).

### Relative Competition Intensity (RCI) of *A. philoxeroides*

**FIGURE 4:**
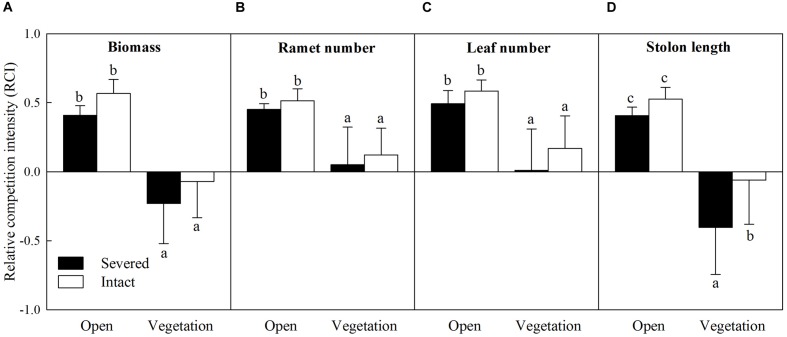
**Effects of experimental treatments on the intraspecific relative competition intensity (RCI) of *A. philoxeroides*.** The intraspecific RCI of biomass **(A)**, ramet number **(B)**, leaf number **(C)** and stolon length **(D)** of the invasive plant *A. philoxeroides* in the apical sections, grown either in open habitats or in vegetative habitats (*J. repens*), and with stolon connections between basal and apical ramets were either intact or severed. Data indicate the means ± SE. Bars sharing the same letter are not significantly different at *P* = 0.05.

The intraspecific RCI of all the growth measures of *A. philoxeroides* were significantly affected by habitat conditions (vegetation), however, those values were not affected by clonal integration except for the intraspecific RCI on stolon length (**Table 2**). The values of the intraspecific RCI of biomass, ramet number, leaf number, and stolon length were significantly lower in vegetative habitats than in open habitats (**Figure 4**). Interestingly, the values of the intraspecific RCI of biomass and stolon length were negative in vegetative habitats (**Figures 4A,D**), indicating that the intraspecific interactions between *A. philoxeroides* individuals changed from competition in open habitats to facilitation in vegetative habitats.

**Table 2:** Two-way ANOVA analyses for the effects of habitat conditions (vegetation) and clonal integration (connection) on the intraspecific relative competition intensity (RCI), and the effects of propagule pressure and clonal integration on the interspecific RCI of the growth measures of the invasive plant *A. philoxeroides* in the apical sections.

Source of variation	df	Error	Biomass (g)	Ramet number^1^	Leaf number^1^	Stolon length (cm)
**Intraspecific RCI**						
Vegetation (V)	1	16	48.46^∗∗∗^	25.89^∗∗∗^	25.92^∗∗∗^	40.95^∗∗∗^
Connection (C)	1	16	3.01	0.74	2.10	4.68^∗^
V × C	1	16	0.01	0.03	0.18	1.14
**Interspecific RCI**						
Propagule pressure (P)	1	16	51.69^∗∗∗^	44.17^∗∗∗^	30.05^∗∗∗^	51.20^∗∗∗^
Connection (C)	1	16	0.70	0.35	0.41	0.46
P × C	1	16	1.00	0.24	0.05	0.02

^1^*Lg, transformed. ^∗^P < 0.05, ^∗∗∗^P < 0.001.*

**FIGURE 5:**
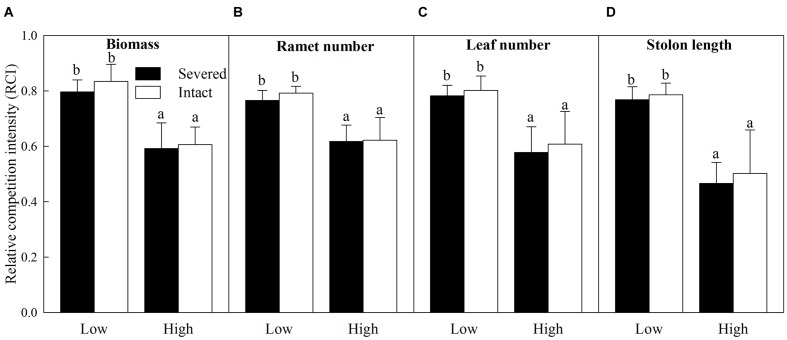
**Effects of experimental treatments on the interspecific RCI of *A. philoxeroides*.** The interspecific RCI of biomass **(A)**, ramet number **(B)**, leaf number **(C)** and stolon length **(D)** of the invasive plant *A. philoxeroides* in the apical sections under either low or high propagule pressure, and with stolon connections between basal and apical ramets were either intact or severed. Data indicate the means ± SE. Bars sharing the same letter are not significantly different at *P* = 0.05.

The interspecific RCI of all the growth measures of *A. philoxeroides* were significantly affected by propagule pressure, whereas those values were not affected by clonal integration (**Table 2**). The values of the interspecific RCI of the four growth measures were significantly lower when propagule supply was high than when it is low (**Figure 5**), suggesting that interspecific competition that vegetation of *J. repens* posed on *A. philoxeroides* became weaker when propagule supply was higher.

### Growth of *J. repens*

Total biomass of *J. repens* vegetation in the apical sections had no significant differences among all the treatments (*F*_4,20_ = 1.15, *P* = 0.21). Biomass in the apical sections of *J. repens* in five treatments (control, low propagule pressure, low propagule pressure with clonal integration, high propagule pressure and high propagule pressure with clonal integration) were 82.68 ± 4.30, 87.18 ± 5.78, 84.02 ± 3.74, 78.59 ± 6.12, and 80.82 ± 4.89 g (means ± SE), respectively.

## Discussion

As more propagules arrive in a new habitat, the probability of successful invasion increases due to either increased propagule numbers or increased frequency of arrival events (Lockwood et al., 2005; Simberloff, 2009). Therefore, propagule pressure may be the primary control parameter for preventing invasions (Lockwood et al., 2005; Liu et al., 2014). In our study, *A. philoxeroides* with high propagule supply grew better and thus probably had higher chance to establish and invade into new habitats than with low propagule supply in both open and vegetative habitats (Lockwood et al., 2009; Liu et al., 2014). In open habitats, high propagule supply increased the growth and clonal propagation (ramet number and stolon length) of *A. philoxeroides* at the expense of reducing the growth of individual plants (**Figure 3**) due to increase in intraspecific competition (intraspecific RCI was relatively high, **Figure 4**). Surprisingly, however, when grown with native vegetation, the enhanced performance of *A. philoxeroides* by high propagule supply did not sacrifice the growth of individual plants. These results support our first hypothesis, suggesting that the role of propagule pressure in the growth and invasion of *A. philoxeroides* population may be more important in vegetative habitats than in open habitats (Liu et al., 2014).

In open habitats, clonal integration significantly improved the growth of *A. philoxeroides* with both low and high propagule supply. This result occurred most likely because the relatively older ramets in the basal sections supported the growth of the interconnected young apical ramets and facilitated the production of new tissue due to acropetal (from basal ramets to apical ramets) translocation of carbohydrates, suggesting that clonal integration plays an important role in exploring new open space and rapid expansion for this invasive plant (Wang et al., 2008; You et al., 2013). Moreover, with low propagule supply, clonal integration also resulted in increased biomass and ramet production of the individual plants, suggesting that clonal integration may be crucial for growth and spread of *A. philoxeroides* in new habitats when propagule pressure is relatively low. However, clonal integration contributed little to the growth and competitive ability of *A. philoxeroides* in vegetative habitats, even with high propagule supply (**Figures 2–5**). This result does not support our second hypothesis, probably because the role of clonal integration in the invasion process of *A. philoxeroides* is occupying open new space and spread, but not increasing competitive ability (Wang et al., 2008; You et al., 2014a).

Without native vegetation, the intraspecific interaction of *A. philoxeroides* was competition, as verified by the positive values of RCI (**Figure 4**). However, in vegetative habitats, there are both intraspecific and interspecific interactions (Mangla et al., 2011; Liu et al., 2014). Under such a habitat condition, the interspecific interaction on *A. philoxeroides* was competition (**Figure 5**, positive values of RCI), whereas the intraspecific interaction on *A. philoxeroides* was facilitation, as demonstrated by the negative values of RCI (**Figure 4**). The shift in the intraspecific interaction on *A. philoxeroides* from competition in open habitats to facilitation in vegetative habitats was also found by Liu et al. (2014), which showed a similar trend of intraspecific interaction on another introduced clonal plant, *Hydrocotyle vulgaris*. The shift in the intraspecific interaction occurred most likely because the native vegetation *J. repens* imposed a severe interspecific competition on *A. philoxeroides*, as shown by the relatively high positive values of RCI (Fajardo and McIntire, 2011). Therefore, the relatively importance of intraspecific and interspecific interactions of *A. philoxeroides* may directly affect the role of propagule pressure in its invasion to native plant community. For example, when propagules of *A. philoxeroides* are introduced into a habitat with dense native vegetation, if the relative effect of intraspecific interaction is lower than interspecific interaction or even shifts to facilitation, as the results showed in this investigation, then high propagule pressure will undoubtedly facilitate the invasion of *A. philoxeroides*. This finding may partially explain why invasions of *A. philoxeroides* are so wide in diverse habitat conditions. Hence, reducing propagule pressure in introduced regions may effectively control the invasion success of this clonal weed into native vegetation, thereby preventing biodiversity loss of native plant communities due to plant invasion (Lockwood et al., 2005; Warren et al., 2012). In the other case, if the relative effect of intraspecific interaction is higher than that of interspecific interaction, then the positive effects of high propagule pressure on invasion success of exotic species may be counteracted by the severe intraspecific competition of individual plants (Liu et al., 2014). In this situation, high propagule pressure will contribute little to the invasion of introduced plants to native plant communities, and controlling the propagule number may not be an effective way to prevent invasion for these species.

Interestingly, total biomass of native vegetation (*J. repens*) was not affected by the presence of *A. philoxeroides*. This result did not accord with our third hypothesis, suggesting that invasion of *A. philoxeroides* in present study did not suppress growth of native plant populations. This is most likely because that competition between apical ramets and native vegetation was asymmetrical because of low density of *A. philoxeroides* in this experiment and their biomass was too small to influence *J. repens* (You et al., 2014a). This observation was supported by the fact that biomass of apical parts of *A. philoxeroides* in vegetative habitats was sharply decreased to less than 35% as compared with that in open habitats. These findings suggest that the propagule pressure (propagule number) examined in this study did not reach or exceed a certain level to influence native vegetation, indicating that *A. philoxeroides* needed more propagules or more time to accumulate enough propagules to establish itself and then invade native plant communities (Lockwood et al., 2009; Simberloff, 2009).

## Conclusion

In conclusion, increased propagule pressure greatly facilitated the growth and potential invasion of *A. philoxeroides*, especially when it grew in vegetative habitats. This is probably due to the shift in the intraspecific interaction on *A. philoxeroides* from competition in open habitats to facilitation in vegetative habitats. Moreover, clonal integration did not affect the growth and competitive ability of *A. philoxeroides* in vegetative habitats, even with high propagule supply, suggesting that clonal integration may be of most important for *A. philoxeroides* to explore new open space and spread, especially when propagule supply was low, but may contribute little to its competitive ability and invasion to native vegetation. Using a control experiment, we show that even a relatively small difference in the number of propagule supply can greatly affect the invasion success of invasive clonal plants, and such an effect also depends on habitat conditions. Furthermore, our study may add support to the argument that high propagule pressure may facilitate invasion (Lockwood et al., 2005; Simberloff, 2009), suggesting that the effects of propagule pressure on the establishment and growth of clonal plants may be an important component of plant risk assessment that is used to identify their potential invasiveness before introduced.

## Author Contributions

Conceived and designed the experiments: W-HY and D-LD. Performed the experiments: W-HY, C-MH, and L-XF. Analyzed the data: W-HY and C-MH. Contributed reagents/materials/analysis tools: W-HY and D-LD. Wrote the paper: W-HY and DLD.

## Conflict of Interest Statement

The authors declare that the research was conducted in the absence of any commercial or financial relationships that could be construed as a potential conflict of interest.
